# Final Results of a Phase I/II Trial of the Combination Bendamustine and Rituximab With Temsirolimus (BeRT) in Relapsed Mantle Cell Lymphoma and Follicular Lymphoma

**DOI:** 10.1097/HS9.0000000000000398

**Published:** 2020-06-08

**Authors:** Georg Hess, Karola Wagner, Ulrich Keller, Paul La Rosee, Johannes Atta, Kai Hübel, Christian Lerchenmueller, Daniel Schoendube, Mathias Witzens-Harig, Christian Ruckes, Christoph Medler, Christina van Oordt, Wolfram Klapper, Matthias Theobald, Martin Dreyling

**Affiliations:** 1 Department of Internal Medicine III (Hematology, Oncology, Pneumology), University Medical Center of the Johannes Gutenberg-University, Mainz, Germany; 2 Department of Hematology, Oncology, Charité - University Medical School Berlin, Berlin Germany; 3 III. Medical Department, Technical University Munich, Munich, Germany; 4 Department of Internal Medicine II (Hematology and Oncology), University Hospital Jena, Germany; 5 Department of Internal Medicine II, Center of Internal Medicine, Johann Wolfgang Goethe-University, Frankfurt, Germany; 6 Department of Internal Medicine I Oncology and Hematology, University Hospital Cologne, Germany; 7 Group Practice of Hematology and Oncology, Münster, Germany; 8 Department of Internal Medicine II, Center of Internal Medicine, Johann Wolfgang Goethe-University, Frankfurt, Germany; 9 Department of Internal Medicine V, University of Heidelberg, Germany; 10 Interdisciplinary Centre for Clinical Trials (IZKS), University Medical Center of the Johannes Gutenberg-University, Mainz, Germany; 11 Division of Hematophathology, Christian-Albrechts-University, Kiel, Germany; 12 Department of Medicine III, Ludwig - Maximilians-University Munich, Germany

## Abstract

In this phase I/II study, we explored the combination of Temsirolimus with Bendamustine and Rituximab (BeRT) in patients with relapsed or refractory (r/r) follicular lymphoma (FL) or mantle cell lymphoma (MCL). Patients with 1 to 3 previous therapies received Bendamustine (90 mg/m^2^, day 1 + 2) and Rituximab (375 mg/m^2^, day 1) with Temsirolimus in doses from 25 to 75 mg in phase I and 50 mg Temsirolimus in phase II, added on day 1, 8, 15 of a 28 days cycle. The primary endpoint of the phase II was ORR at the end of treatment. Overall, 39 (29 MCL, 10 FL) patients were included. Median age was 71 years and median pretreatment number was 2. Grade 3/4 non-hematologic adverse events were rare and included hyperglycemia in 3 patients (7%) and angioedema in 2 patients (5%). Infectious complications grade 3/4 were observed in 9 patients (23%). Hematologic grade 3/4 events included leukopenia in 22 (56%), neutropenia in 18 (46%), lymphopenia in 16 (41%) and thrombocytopenia in 14 patients (36%). An objective response (best response) was observed in 33/39 patients (89%; 24 MCL (89%) and 9 FL (90%)), including 14 CR (38%; 12 MCL (36%) and 2 FL (20%)). Median PFS is 1.5y for MCL and 1.82 years for FL, and median OS has not been reached for either entity. This data demonstrates promising efficacy of Temsirolimus in r/r MCL and FL with acceptable toxicity. The BeRT regimen may be used as a treatment option for both entities.

## Introduction

Although improvements in first line and second line treatments of patients with follicular lymphoma (FL) or mantle cell lymphoma (MCL) have been achieved, these lymphomas still are considered incurable with available conventional strategies.^1–4^ First line results to chemoimmunotherapy give reasonable results, however, if repeated responses typically deteriorate over subsequent treatment lines. New hope came from the introduction of targeted agents. Among these new options inhibitors of the mammalian Target of rapamycin (mTOR) have been explored in different malignant diseases.^5^ mTOR is a master switch of protein translation and a key element of the PI3K/AKT/mTOR pathway. Activation of mTOR markedly enhances the mRNA translation of an important group of growth-related proteins that include cyclin D1, c-MYC, and hypoxia-inducible factor 1α. Functionally, mTOR activity increases cellular proliferation as well as growth- and survival-pathways and inhibits autophagy. mTOR also regulates the translation of other proteins that are potential oncogenes contributing to lymphomagenesis (eg, cyclin A, c/EBPβ, and survivin).^6,7^

Clinically, in lymphoma mTOR inhibitors were primarily evaluated in MCL based on constitutional overexpression of cyclin D1. Phase II trials with Temsirolimus, a derivative of Rapamycin, demonstrated responses in up to 40% of patients with r/r MCL, and other mTOR-inhibitors have shown similar results.^8–10^ A randomized three-arm phase III trial showed dose dependent superiority of ORR and PFS for Temsirolimus compared to alternative chemotherapeutic options in rr MCL,^11,12^ subsequently resulting in approval. However, complete remissions and long-term remissions are rarely observed with single agent treatment. Beyond MCL, promising activity of Temsirolimus has also been demonstrated in other lymphoproliferative diseases.^13–16^ In a phase II study by Smith et al^15^ evaluating Temsirolimus at a weekly dose of 25 mg in patients with diffuse large B-cell lymphoma an overall response rate of 28% was observed and even more impressive, in r/r FL 54% of patients achieved an objective response. However, long term treatments with mTOR inhibitors are associated with cumulative toxicities, a treatment limitation could therefore be attractive.

The combination of Temsirolimus with Rituximab for MCL has already been explored, with a notable increase of the overall (59%) and complete remission rates (19%) as well as progression free survival in 71 patients.^17^ In addition, the combination of Temsirolimus with classical cytotoxic agents, for example, cladribine^18^ demonstrates acceptable tolerability and promising efficacy. Consequently, we aimed to evaluate the triplet of chemotherapy, monoclonal antibody and Temsirolimus. Bendamustine combined with Rituximab (BR) is a worldwide accepted regimen for MCL and FL with a well-defined safety profile and^19–21^ preclinical evidence supports the combination with Temsirolimus.^22^

Therefore, we initiated a phase I/II trial to firstly explore the maximum tolerated dose of the Bendamustine, Rituximab, and Temsirolimus (BeRT) combination and secondly evaluate the efficacy of this regimen. Here we report the final analysis of this trial including patients from the phase I population and the phase II cohort.

## Results

### Patients

A total of 39 patients, 28 males and 11 females, entered the phase I/II trial between 02/2010 and 02/2015. Thereof, 15 patients (11 MCL and 4 FL) were included in the phase I part of the trial. The characteristics of all enrolled patients are outlined in Table 1. In brief, median age was 71 years. 29 patients had relapsed MCL and 10 had FL. The median number of prior regimens was 2 (range 1–3), and all patients had received Rituximab treatment prior. Nine patients were pretreated with Bendamustine and 6 patients had previously received a high dose therapy. 35% of patients had at partial response or better to their last treatment line, whereas 60% were refractory (41% with progressive disease).

**Table 1:**
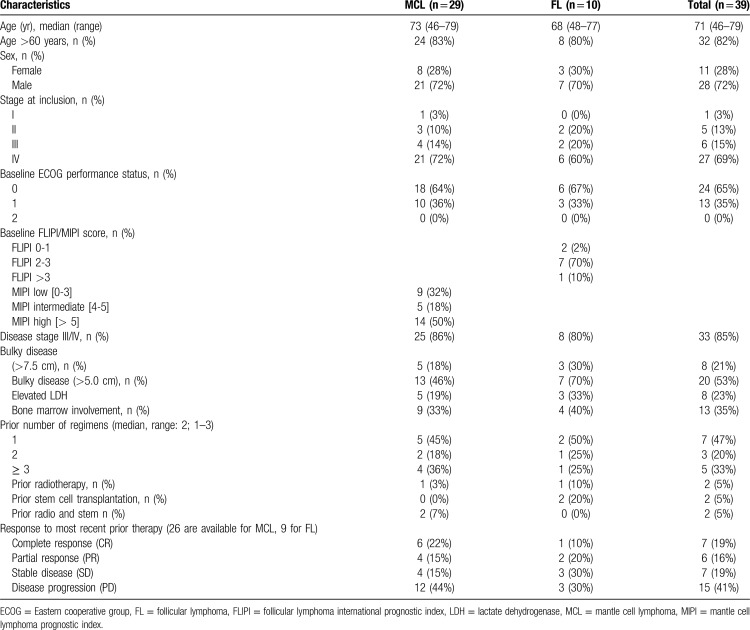
Patient Characteristics at Inclusion into Trail.

### Temsirolimus doses, completion of treatment and relative dose intensity

In phase I each 3 patients were treated at the Temsirolimus 25 mg and 50 mg dose level, 6 patients were treated at the 75 mg dose level. All 27 patients in phase II were treated at the 50 mg dose level, therefore 30/39 patients were treated at the recommended phase II dose.

In total 133 of 156 pre-planned cycles were started (85%) and 101 cycles were completed as planed (65%). The median number of treatment cycles received was 4 and 27 patients entered the last cycle period.

The cycle length was on average 31 days (27 to 44 days), in detail cycle 1: 30.1 days (27 to 42 days), cycle 2: 31.4 days (27 to 42 days), and cycle 3: 31.7 days (27 to 44 days). Taken together, most cycles were given without any delay, reflected by a median cycle length of 28 days. Proportional the total number of cycles started, the following dose intensities were observed: Rituximab 100%, Bendamustine 99.6% and Temsirolimus 96.8% (Table 2), demonstrating that overall treatment was well tolerated.

**Table 2:**
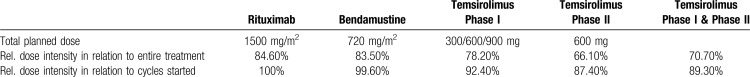
Dose Adherence.

### Adverse events (AE)

In all 39 patients who received study treatment at least once, the following hematologic AEs (any grade) were observed (number of patients (percentage)) (Table 3): leukopenia in 28 (72%), thrombocytopenia in 25 (64%), neutropenia in 20 (51%), lymphopenia in 16 (41%) and anemia in 11 patients (28%). Grade 3/4 hematologic adverse events in all treatment levels and comprising all cycles occurred as follows: leukopenia in 22 (56%), neutropenia in 18 (46%), lymphopenia in 16 (41%) and thrombocytopenia in 14 patients (36%) (Table 3). Taken together, 7 patients required at least one erythrocyte transfusion and 3 patients needed at least one platelet transfusion, G-CSF was used in 12 patients.

**Table 3:**
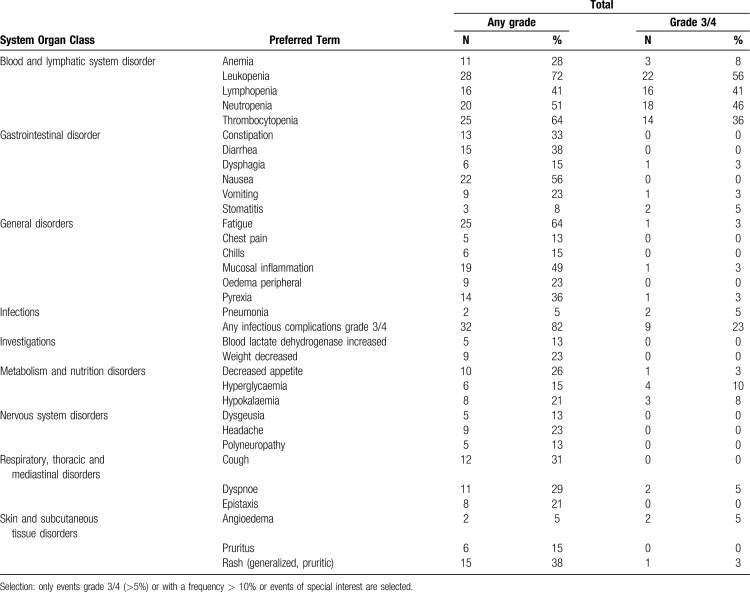
Adverse Events, Any Grade and Grades 3/4.

The most frequent non-hematologic treatment emergent adverse events of any grade were fatigue in 25 (64%), nausea in 22 (56%), mucositis in 19 (49%), diarrhea and rash in 15 (38%), pyrexia in 14 (36%), constipation in 13 (33%), and cough in 12 (31%) patients. Grade 3/4 non-hematologic adverse events were rare and included hyperglycemia in 4 patients (10%) and angioedema in 2 patients (5%). Any infectious complications grade 3 or 4 occurred in 9 patients (23%) including stomatitis, mucositis, and pneumonia.

### Efficacy – response rates

Responses were evaluable in 37/39 patients enrolled (Table 4). The objective response rate for all patients was 89%, with rates of 89% and 90% for MCL and FL, respectively. Complete remissions were observed in 38% of the entire group, and 44% and 20% of MCL and FL patients, respectively. Stable disease was observed in 11% of the patients, 3 with MCL and 1 patient with FL. If the rate of SD is added to CR/PR, it would result in a clinical benefit rate of 100%.

**Table 4:**
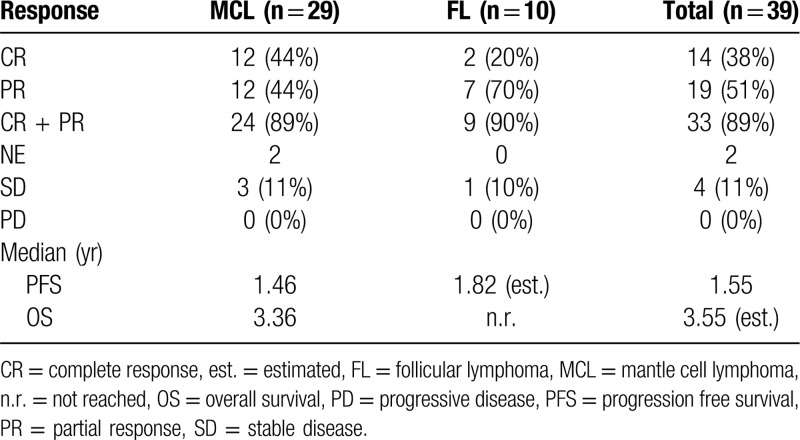
Response Rates and Progression-free and Overall Survival.

Responses were observed early: out of 28 patients evaluated after 2 cycles, 26 patients had already a partial remission and 2 complete responses were observed at this time point.

Progression free survival and overall survival.

At the time of analysis median follow up of all patients was 2.7 years, with the longest follow up of 4.4 years.

**Figure 1:**
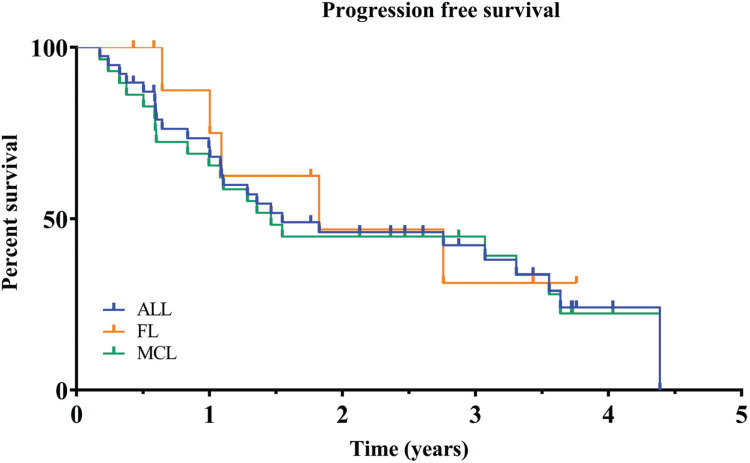
**Kaplan-Meier curves of progression-free survival for all patients enrolled**. Results for time-to-event end points were analyzed according to Kaplan-Meier estimator. Median Progression free survival for the entire cohort was 1.6 years. PFS = Progression free survival.

Median progression free survival for the entire cohort was 1.6 years (95% CI: 1.08 to 3.55), 1.5 years (95% CI: 0.84 to 3.55) for patients with MCL and 1.82 years (95% CI: 0.64 to unknown) for patients with FL (Fig. 1).

**Figure 2:**
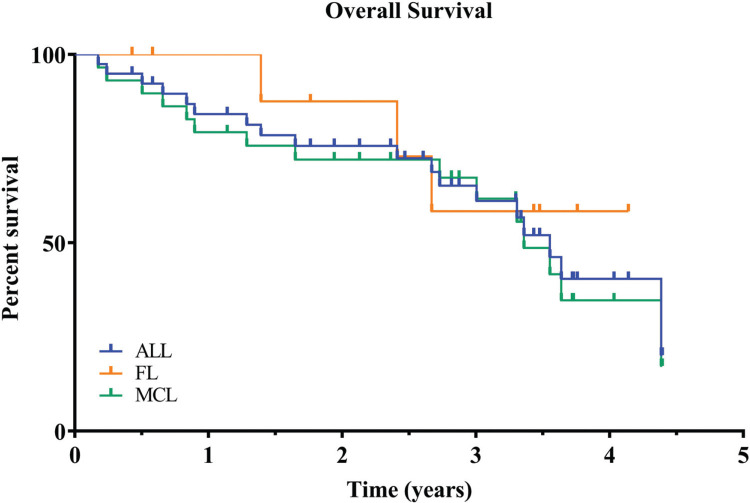
**Kaplan-Meier curves of overall survival for all patients enrolled**. Results for time-to-event end points were analyzed according to Kaplan-Meier estimator. After 3 years of follow up 57% of all patients remain alive. OS = Overall survival.

Median overall survival has not been reached for either the entire group of patients or any subgroup. After 3 years of follow up 57% of all patients remain alive (Kaplan-Meier estimate), 56% for MCL and 58% for FL (Fig. 2).

## Discussion

Novel treatment options have already impacted current treatment algorithms. Especially in MCL several novel agents have been successfully introduced, particularly BTK-inhibitors have significantly changed the current treatment and trial strategies.^23–25^ In parallel, other drugs have been successfully tested, for example, Temsirolimus, Lenalidomid or Bortezomib and recently Venetoclax,^26–31^ however, their optimal placement within treatment sequencing is less clear. In FL Idelalisib, a PI3K inhibitor, has been approved and other agents of this class are on their way to clinical practice. Furthermore, Lenalidomide in combination with Rituximab is a potential alternative relapse treatement.^32^

Chemotherapy, although cumbersome, has been shown to overcome resistance to novel agents^33–35^ and in addition, offers a treatment free window for patients in contrast to treatment until progression with targeted agents. Enhancement of chemotherapy with novel agents might therefore be a rational strategy to deepen responses and therefore prolong remission duration and, at the same time, limit treatment duration. In our trial we evaluated the combination of BR with Temsirolimus. Overall, we observed promising response rates. 89% of MCL and 90% of FL patients showed at least a partial response and interestingly a substantial number of CRs (12 MCL (44%); 2 FL (20%)) were observed. Keeping in mind that BeRT was intended to be a short duration treatment (only 4 cycles were given), these response rates are encouraging. Considering that 60% of the study population had not responded to their individual last treatment line, this underlines that the combination is able to overcome drug resistance in a substantial proportion of patients. We noted a promising duration of progression free and overall survival. Duration of response was longer in patients with FL; however, this difference can be expected due to the more indolent nature of this lymphoma subtype.

If our results are compared to chemo-immunotherapies used in second line of MCL, they compare favorable to treatments such as Fludarabin, Cyclophosphamid and Rituximab or Gemcitabine. Oxaliplatine and Rituximab and compare well to other Bendamustine-containing combinations.^19,36–38^ In these trials, the majority of patients had only received 1 prior treatment line and importantly had been Rituximab naïve in the majority of cases, which reflects a more adverse risk profile in our trial. The trial of Visco et al,^39,40^ BR was combined with Cytarabinoside and high responses and promising response durations were observed. However, this treatment showed to harbor significant toxicities, for example, platelet transfusion are required in up to 60% to 70% of patients. Thus, this regimen is restricted to a different population of patients as used in our trial. Zaja and colleagues evaluated BR plus Lenalidomide^41^ and it showed comparable response rates and remission durations – however, the treatment duration was up to 2 years for responding patients, which limits comparability. Another trial evaluating 6 cycles of a similar combination and maintenance for 8 months in first line, demonstrated a high CR rate of 64%^42^ and promising median PFS. But substantial side effects were found as well as a high rate of secondary primary malignancies, limiting its value especially in younger patients. In comparison to single targeted agent, with all precautions taken, only results of BTK inhibitor treatment compares favorable to our results^24,25^ which is reflected in current recommendations. Consequently, it would be of great value to have data available in patients with BTK-failure. Some agents have shown preliminary but frequently moderate efficacy in this situation. Lenalidomide was able to induce remission in 30%^32^ but duration of response is not satisfying with only 2 months in median. Venetoclax seems to induce remissions to a higher extent; however, again PFS is probably short.^43^ Therefore, the combination of various novel agents may become a preferred strategy, foe example, the combination of Ibrutinib and Venetoclax,^44^ but if any of these approaches are moved to frontline, the question of treatment failure will reappear. As Bendamustine combinations have been shown to be effective in a substantial number of patients in BTK-failure,^33,45^ our data might offer an alternative option in this scenario.

In FL, novel agents only slowly enter the clinical arena. Idelalisib is used with precautions only due to its toxicity profile. In r/r indolent lymphoma patients a median PFS of 11 months was achieved, but the data cannot be compared to our results, as the selection criteria differed significantly. The results of Copanlisib seem equivalent.^3^ The combination of Lenalidomide with chemotherapy has been evaluated in various trials^46,47^ and recently the results of a randomized trial comparing Lenalidomide and Rituximab to Rituximab were shown. PFS was 39 months for patients with FL, however, there is substantial differences in patient and treatment characteristics, compared to our trial. Other drugs gave rather disappointing results in relapsed FL and the number of available options for remains limited.^48,49^ Despite our low patient numbers, we find high response rates and reasonable PFS, importantly better than in the individual prior treatment line (89% with BERT and 30% with previous line) and therefore believe our data proves to be promising for further evaluation.

Further work is needed to more precisely define the future role of BeRT^15^ both in MCL and FL. As the treatment landscape is evolving rapidly and there are limited options for randomized comparisons, real world treatment data bases will be helpful to give additional information, which we aim to analyze in the future.

The tolerability of the BeRT treatment was acceptable, with limited need for transfusion and fatigue being the dominant side effect. Especially in the phase II part of the trial, after establishment of the MTD, the rate of side effects remained reasonable and most patients were able to complete all 4 cycles of the treatment. However, we did observe that the 3rd dose of Temsirolimus was occasionally dropped (in 32.4% of FL cases and in 25.5% of MCL cases) due to moderate thrombocytopenia which resulted in a relative dose intensity of 87% for Temsirolimus. As there was no obvious loss of response in patients with significant thrombocytopenia, this could be a reasonable approach to maintain cycle intervals stable.

Therefore, given the high response rates, the short treatment duration and the acceptable side effects profile, we believe that the results presented here may help to establish the BeRT regimen as an alternative back up option especially for patients with MCL.

## Methods

### Conduct of the trial

According to national regulations, the study was approved by a harmonized approach of the appropriate Ethic's Committees and conducted in accordance with Good Clinical Practice guidelines. Patients were required to sign informed consent prior to any study related procedures. Trial conduct was supported by the IZKS (Interdisciplinary Center for Clinical Trials) of the University Medical Center Mainz. The trial is registered on clinicaltrials.gov (NCT01078142).

### Study design

This was a prospective multicenter, phase I/II, open-label study of established regimen for Bendamustine and Rituximab in combination with Temsirolimus. In the phase I part of the study a MTD was established, which was defined as published before.^50^

### Patients

For this analysis, patients with r/r follicular (Grade I-IIIA) or mantle cell lymphoma after one to 3 prior lines of therapy were considered eligible. In addition, diagnosis of MCL had to be confirmed by either Cyclin D1 overexpression (immune-histochemistry) or proof of chromosomal translocation t(11;14). A central pathology review was mandatory.

### Main eligibility criteria

Patients with MCL or FL, at least one prior line of treatment including Rituximab, no curative options available and need for treatment in FL. Adult patients had to have an Eastern Cooperative Group (ECOG) performance status of 0–2, an appropriate hematopoietic reserve (absolute neutrophil count ≥1500/μL; platelets ≥75,000/μL) as well as liver (aspartate aminotransferase (AST) and alanine aminotransferase (ALT) <2.5 times ULN, total bilirubin <1.5 times the upper limit of normal (ULN)) and renal function (creatinine clearance > 50 ml/min). Disease manifestation had to be measurable (≥ 1 disease lesion >1.5 cm × 1.0 cm or bone marrow infiltration). No prior allogeneic stem cell transplantation was allowed.

### Treatment regimen

The BeRT regimen consisted of Bendamustine given at a dose of 90 mg/m^2^ i.v. day 1 and 2 every 4 weeks, Rituximab 375 mg/m^2^ i.v. was given on day 0 or 1. After establishment of a maximum tolerated dose in the first part of this study, Temsirolimus was given at a dose of 50 mg i.v. on day 2, 8, 15 of a 4 week cycle. A total of 4 treatment cycles was planned, as this had been shown to be efficacious and safe in preceding trials.^19,38^ G-CSF use was up to the investigator's discretion.

Adverse events were graded according to the National Cancer Institute's Common Terminology Criteria for Adverse Events (CTCAE) version 4.03.

### Evaluations

All patients starting study treatment in phase I and phase II were included in the safety population, and efficacy analyses were performed in all patients enrolled on an intent-to-treat (ITT) basis.

Response to treatment was defined according to the 2007 international response criteria for non Hodgkin lymphoma.^50^ Initial staging included CT-scans of neck, chest, abdomen and pelvis and radiological evaluation of all affected regions. Re-staging was scheduled after 2 and 4 cycles and every 3 months for the first 12 months, followed by 6-months intervals thereafter. PFS was defined as time from treatment start to date of relapse or refractoriness, disease progression or death, or censored at the last tumor evaluation date. OS was measured as time from first dose to date of death or censored at the last date of patient contact. After progression or the initiation of a new therapy, patients were followed with respect to survival.

### Objectives and statistical analysis

Phase I defined the MTD of BeRT whereas efficacy was the primary aim of the phase II part of the study.

The primary endpoint was the objective response rate (number of patients with PR and CR) in patients with mantle cell or follicular lymphoma treated at the maximum tolerated dose of BERT. Secondary endpoints were the rate of side effects observed, overall response rate (CR, PR), progression free survival (PFS), duration of response, time to subsequent lymphoma therapy, treatment free interval and overall survival (OS). Due to the explorative character, no formal sample size calculation was performed. However, Wald's sequential probability ratio test was applied to exclude that the number of no-responders exceeded a critical value.

Results for time-to-event endpoints were analyzed according to Kaplan-Meier estimator. SAS Version 9.4. (SAS Institute, Cary, NJ) was used for all calculations.

## Acknowledgments

We thank the patients, their families, the clinical research staff at the various clinical sites and the IZKS for their contribution to this work. We thank A. Ohler for her assistance on the preparation of the manuscript. This trial was conducted within the “German Lymphoma Alliance”, formerly “German low grade lymphoma study group (GLSG)”.
